# Multifaceted Assessment of Chronic Gastritis: A Study of Correlations between Serological, Endoscopic, and Histological Diagnostics

**DOI:** 10.1155/2011/631461

**Published:** 2011-07-10

**Authors:** Toshitatsu Takao, Takeshi Ishikawa, Takashi Ando, Madoka Takao, Tsuguhiro Matsumoto, Yutaka Isozaki, Mika Okita, Yasuyuki Nagao, Hirokazu Oyamada, Keiichi Yokoyama, Atsushi Tatebe, Kazuhiko Uchiyama, Osamu Handa, Tomohisa Takagi, Nobuaki Yagi, Satoshi Kokura, Yuji Naito, Toshikazu Yoshikawa

**Affiliations:** ^1^Department of Molecular Gastroenterology and Hepatology, Graduate School of Medical Science, Kyoto Prefectural University of Medicine, 465 Kajii-cho, Kamigyo-ku, Kyoto 602-8566, Japan; ^2^Department of Gastroenterology, Matsushita Memorial Hospital, 5-55 Sotojima-cho, Moriguchi city, Osaka 570-8540, Japan; ^3^Department of Pathology, Matsushita Memorial Hospital, 5-55 Sotojima-cho, Moriguchi, Osaka 570-8540, Japan

## Abstract

* Aim*. Chronic gastritis was assessed serologically, endoscopically and histologically to identify correlations between these methods. *Methods*. Subjects comprised 319 patients who had provided informed consent. Serological assessment of chronic gastritis was based on the pepsinogen test method. Endoscopic gastritis and histological gastritis were assessed and scored according to the Kimura-Takemoto classification system and the updated Sydney classification system respectively, and correlations between these three methods were studied. *Results*. Pepsinogen I/II ratio showed a significant correlation to the extent of mononuclear cell infiltration of the gastric corpus. When histological gastritis was divided, on the basis of the distribution of mononuclear cell infiltration, into gastritis limited to the antrum and corpus gastritis, these types were distinguished with high accuracy using a pepsinogen I/II ratio of 3 as the cutoff. A good correlation was also seen between pepsinogen I/II ratio and development of atrophy in endoscopic gastritis, where groups with and without advanced atrophy were also distinguished with high accuracy using a cutoff value of 3. *Conclusion*. Significant correlations exist between serum pepsinogen levels, endoscopic gastritis, and histological gastritis. Pepsinogen I/II ratio allows prediction of the existence of endoscopic gastritis and histological gastritis, or the extent of their development, with high accuracy.

## 1. Introduction

Measurements of the serum levels of pepsinogens (PGs) are widely used to screen for gastric cancer [1, 2]. PG is immunologically classifiable as PG I or PG II, where PG I is primarily secreted into the gastric lumen by the chief cells of the fundic glands and PG II is secreted by the cardiac glands, pyloric glands, and duodenal glands in addition to the fundic glands. PG is present, although in minute levels, in the blood. Measurements of the serum levels of PG I or the ratio of PG I to PG II are well-known nonendoscopic tools for diagnosing atrophic gastritis [3–5]. Samloff et al. reported that atrophic gastritis can be diagnosed on the basis of decreases in serum PG I level and PG I/II ratio [6]. This is referred to as “serologic biopsy”. In a chromoendoscopic study using Congo red, Miki et al. reported a high correlation in stepwise decreases in serum PG I level and PG I/II ratio to increases in the atrophic gland border associated with gastric mucosal atrophy [3]. Several reports have also described correlations between histological gastritis and serum PG levels [7–10], but the available data describing variations in serum PGs with histopathological features of gastritis are limited and somewhat controversial. In children undergoing endoscopy and antral biopsy, a positive correlation has been reported between serum PG I values and the severity of antral inflammatory cell infiltration [7]. However, amongst healthy adult volunteers with gastritis, no correlation between serum PGs and antral inflammation was observed, though significant correlation with corpus inflammation scores was seen [8]. Knight et al. reported that levels of serum PG I decreased with progression from an antral predominant pattern, through pangastritis to a corpus predominant pattern of gastritis [9].

Chronic gastritis can be assessed serologically, endoscopically, and histologically, and these methods are widely used in clinical settings. However, detailed, multifaceted studies of the correlations and points of correspondence between these methods have yet to be undertaken. Multifaceted understanding to chronic gastritis is far from comprehensive. Therefore, we aimed to examine correlations between serological diagnosis based on serum PG levels, endoscopic diagnosis of gastritis based on the Kimura-Takemoto classification system [11], and histological diagnosis of gastritis based on the updated Sydney classification system [12].

## 2. Methods

Subjects comprised 319 patients who had been examined for epigastric symptoms at Matsushita Memorial Hospital between April 2006 and March 2007. The study was approved by the Ethics Committee of Matsushita Memorial Hospital, and each patient gave written informed consent. Patients who were taking oral acid-inhibiting drugs (H2 receptor antagonists or proton pump inhibitors), who had previously undergone *Helicobacter pylori* (Hp) eradication therapy, or who had a history of gastric surgery were excluded. Blood samples were taken while patients were fasting, serum PG levels were determined by Chemiluminescent enzyme immunoassay, and serum Hp antibody titer was determined by enzyme immunoassay (E Plate; Eiken, Tokyo, Japan).

Endoscopy was performed by an endoscopist in the Department of Gastroenterology at Matsushita Memorial Hospital. Endoscopic gastric mucosal atrophy was classified according to the Kimura-Takemoto classification system, using the following scores: absence of any atrophy, 0 point; closed-type gastritis, 1 point; open-type gastritis, 2 points. Diagnosis was made by the endoscopist who performed an examination and the first author, and any differences in opinion were resolved by consensus. For histological assessment of gastritis, biopsies were taken from two locations comprising the antral greater curvature and the greater curvature of the gastric corpus during endoscopy, and specimens were diagnostically analyzed by two pathologists at Matsushita Memorial Hospital. Prior to diagnostic analysis, the pathologists held a discussion using a schema of the parameters (mononuclear cells, neutrophils, atrophy, Hp, intestinal metaplasia (IM)) of the updated Sydney classification system and agreed on a grading procedure. In this study, grades of normal, mild, moderate, and marked findings were given scores of 0, 1, 2, or 3 point, respectively.

Statistical analysis involved the use of SPSS version 11 (SPSS, Chicago, ILL, USA) to compare ≥3 groups using analysis of variance (ANOVA) and to compare pairs by the Fisher Protected Least Significant Difference test. The relationship between two variables was represented using the Pearson correlation coefficient.

## 3. Results

Patient characteristics are given in Table 1. Mean age was 56.5 years (range, 12–94 years), and serum Hp antibody-positive rate was 69.0% (220/319).

Results for the parameters in this study are as follows.

### 3.1. Correlation between Serum PG Levels and Histological Gastritis

Analysis of the correlation between serum PG I and II levels and PG I/II ratio to histological parameter scores based on the updated Sydney classification system revealed that PG I/II ratio showed a stronger correlation to histological findings than did serum PG I or II levels (not shown).  Table 2 shows the correlation between PG I/II ratio and histological parameters by gastric location (antrum and corpus), revealing a significant (*P* < 0.05) correlation between all parameters and PG I/II ratio. This included significant correlations to mononuclear cell infiltration and neutrophil infiltration of the gastric corpus (*r* = −0.389 and *r* = −0.442, resp.). The correlation coefficient of mononuclear cell infiltration of corpus (*r* = −0.442) was stronger than that of another. 

**Figure 1:**
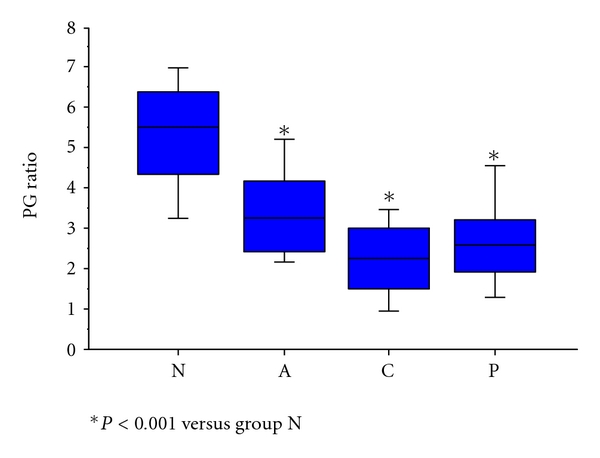
Correlation between serum pepsinogen I/II ratio and topography of gastritis determined by the extent of mononuclear cell infiltration: PG I/II ratio was significantly lower in A, C, and P group than in N group. N: normal or mild gastritis; A: antrum-predominant gastritis; C: corpus-predominant gastritis; P: pangastritis: horizontal bar, median; box: 25th–75th interquartile range: vertical lines: range of values.

**Figure 2:**
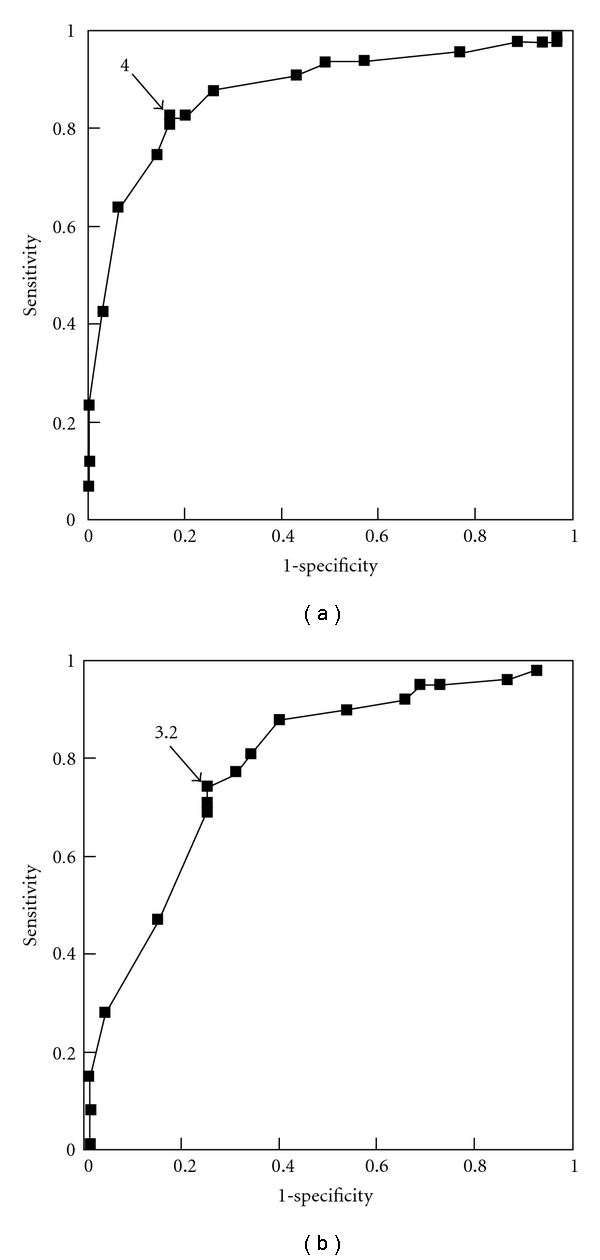
Receiver operator characteristic analysis in the diagnostic capability of histological gastritis by serum pepsinogen I/II ratio. (a) Discrimination of normal or mild gastritis (N Group) from other patterns of gastritis (A, C and P Group). (b) Discrimination of without-corpus gastritis (N and A Group) from with corpus gastritis (C and P Group).

Next, histological gastritis was classified into four groups based on the extent of mononuclear cell infiltration in the antrum and gastric corpus (Table 3): normal or mild gastritis (N); antrum-predominant gastritis (A); corpus-predominant gastritis (C); pangastritis (P). Serum Hp antibody-positive rates for each group were 37.1%, 84.4%, 78.3%, and 86.7%, respectively. Comparison of PG I/II ratio between these four groups revealed that, compared to the N group, PG I/II ratio was significantly lower in the other three groups (Figure 1). Histological gastritis was divided into “no gastritis” (N group) and “gastritis” (A, C, and P groups), and receiver operating characteristic (ROC) analysis of gastritis diagnosis by PG I/II ratio revealed an optimal PG I/II ratio cutoff of 4.0 for a diagnosis of histological gastritis, with 82.6% sensitivity and 82.9% (Figure 2(a)). Upon subsequent division into “two groups without corpus gastritis” (N and A groups) and “two groups with corpus gastritis” (C and P groups), ROC analysis of the diagnostic capability by PG I/II ratio revealed an optimal PG I/II ratio cutoff of 3.2 for the diagnosis of corpus gastritis, with 73.6% sensitivity and 74.6% specificity (Figure 2(b)).

### 3.2. Correlation between Serum PG Levels and Endoscopic Gastric Mucosal Atrophy

**Figure 3:**
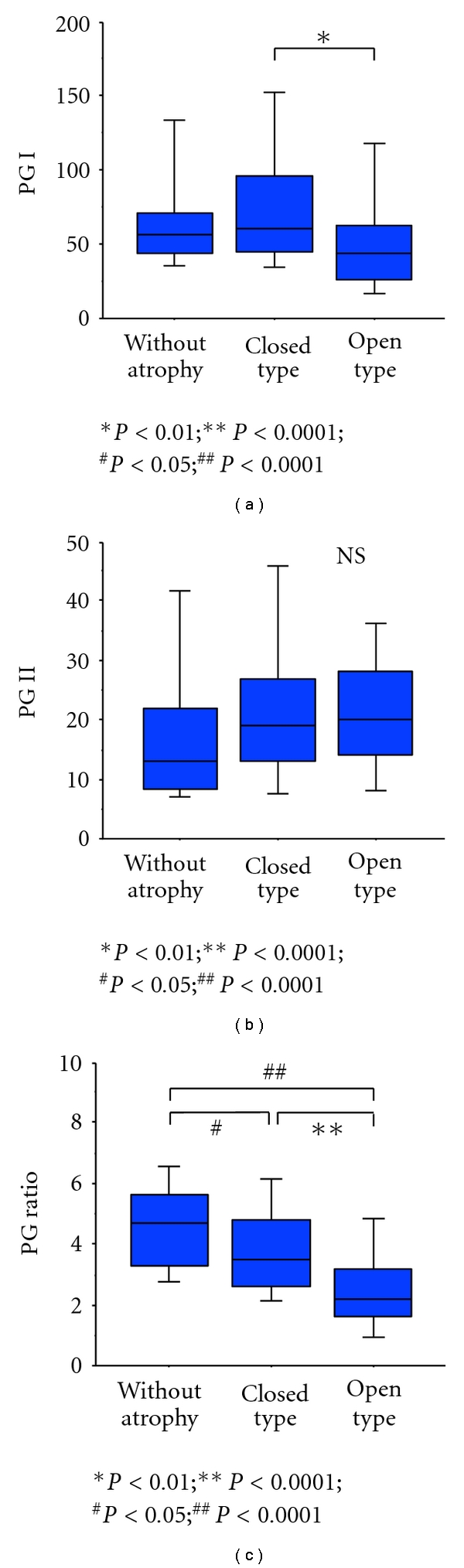
Correlations between serum pepsinogen levels and topography of gastric mucosal atrophy determined by endoscopy. PG I/II ratio decreased significantly as endoscopic gastric mucosal atrophy progressed. Horizontal bar: median; box: 25th–75th interquartile range; vertical lines: range of values; NS: not significant.

Examination of serum PG levels and endoscopic gastric mucosal atrophy revealed serum PG I levels were significantly lower in open-type gastritis compared to the closed-type. PG I/II ratio also decreased significantly as endoscopic gastric mucosal atrophy progressed. No significant differences in serum PG II levels were seen between groups (Figure 3).

**Figure 4:**
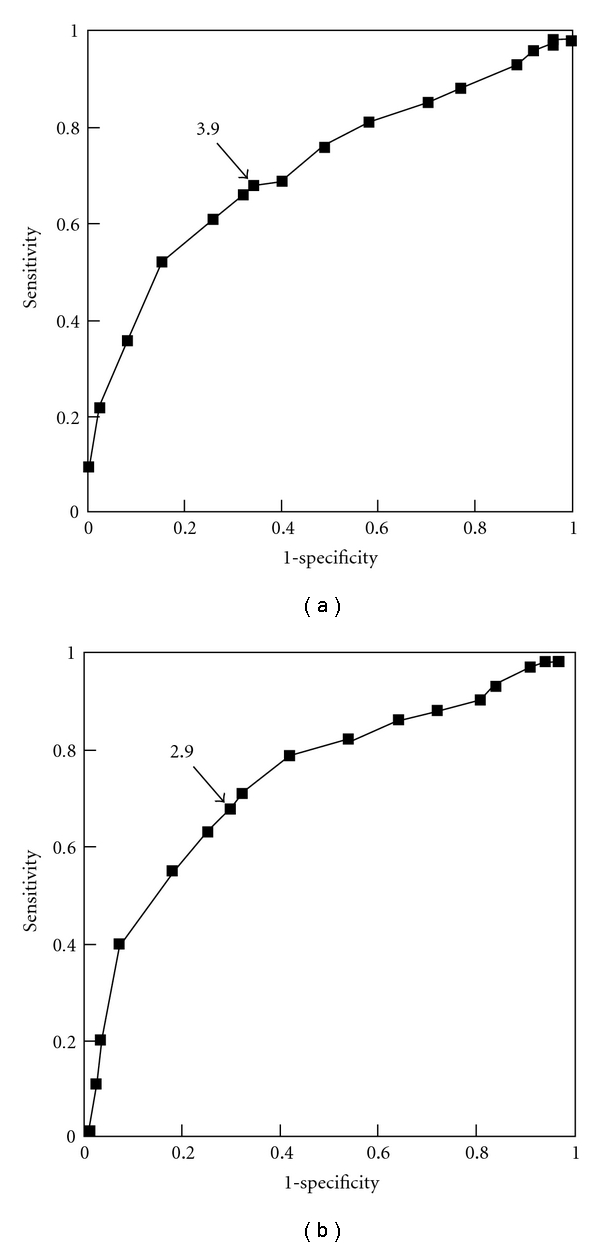
Receiver operator characteristic analysis in the diagnostic capability of endoscopic gastric mucosal atrophy by serum pepsinogen I/II ratio. (a) Discrimination of with endoscopic findings of atrophy from without endoscopic findings of atrophy. (b) Discrimination of without advanced atrophy from with advanced atrophy (open type).

Endoscopic gastric mucosal atrophy was divided into a “group without atrophy” and “groups with atrophy” (closed or open type), and ROC analysis of the diagnostic capability by PG I/II ratio revealed an optimal PG I/II ratio cut-off of 3.9 for distinguishing between presence and absence of endoscopic gastric mucosal atrophy, with 68.0% sensitivity and 66.0% specificity (Figure 4(a)). Upon subsequent endoscopic division into “groups without advanced atrophy” (no gastric mucosal atrophy, closed type) and a “group with advanced atrophy” (open type), ROC analysis of the diagnostic capability by PG I/II ratio revealed an optimal PG I/II ratio cut-off of 2.9 for distinguishing between the presence or absence of advanced atrophy, with 67.9% sensitivity and 70.4% specificity (Figure 4(b)).

### 3.3. Correlation between Endoscopic Gastric Mucosal Atrophy and Histological Gastritis

**Figure 5:**
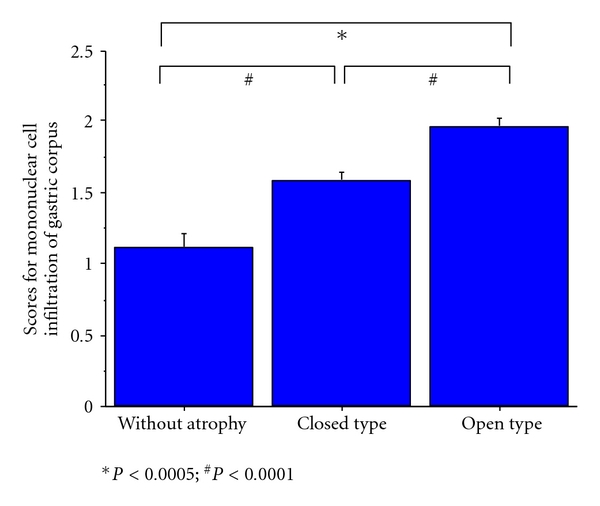
Correlation between scores for mononuclear cell infiltration of the gastric corpus and topography of gastric mucosal atrophy determined by endoscopy. There was a strong correlation between endoscopic gastric mucosal atrophy scores and scores for mononuclear cell infiltration of the gastric corpus.

Analysis of endoscopic gastric mucosal atrophy scores and the Sydney classification-based histological parameter scores by site revealed a significant correlation between endoscopic gastric mucosal atrophy scores and scores for mononuclear cell infiltration of the gastric corpus (*r* = 0.459) (Table 4; Figure 5). Other parameters, including neutrophil infiltration of the gastric corpus (*r* = 0.382), mucosal atrophy of the gastric corpus (*r* = 0.390), and intestinal metaplasia of the antrum (*r* = 0.348), showed significant but weak correlations to endoscopic gastric mucosal atrophy

## 4. Discussion

The concept of chronic gastritis has long been used in the absence of strict definitions, resulting in confusion in the clinical setting. However, with the advent of Hp in the 1980's, chronic gastritis has come to be regarded as a condition in which atrophy progresses with repeated loss and regeneration of the gastric mucosa due to chronic histological inflammation associated with persistent Hp infection [13]. The Sydney system was created in an attempt to more objectively assess chronic gastritis using a visual scale based on the concept of infectious gastritis caused by Hp infection [14, 15], and the updated version [12] is now being used internationally. Although histopathological assessment is widely used in the Sydney system, endoscopic classifications have not yet become widespread, whereas, in Japan, the Kimura-Takemoto endoscopic classification system [11] for chronic gastritis, which focuses on atrophic changes, is now also widely used. 

In a study on correlations between chronic gastritis and PG levels, Samloff et al. [6] measured serum PG I and PG II levels in 1982 and reported that atrophic gastritis could be diagnosed on the basis of decreased PG I levels and PG I/II ratio. Serum PG levels have come to be used as serological markers of atrophic gastritis. With the subsequent advent of Hp and publication of the Sydney system, several reports have been issued on the use of this histological assessment to study correlations between serum PG levels and histological gastritis, and correlations with inflammation of the gastric corpus [8] and atrophy of the gastric corpus [9] have been identified. 

Endoscopy is also prevalent in Japan, and numerous reports have examined correlations between serum PG levels and endoscopic gastritis. Miki et al. reported a high correlation in stepwise decreases in serum PG I level and PG I/II ratio to increases in the atrophic border associated with endoscopic gastric mucosal atrophy [3]. A chromoendoscopic study using methylene blue also showed a high correlation between intestinal metaplasia and serum PG levels [16]. However, no detailed studies on correlations between serum PG levels, endoscopic gastritis, and histological gastritis have yet been reported. 

This study first examined whether any correlations exist between serum PG levels and any of the histological assessment parameters in the updated Sydney classification, revealing significant correlations between PG I/II ratio and neutrophil and mononuclear cell infiltrations of the gastric corpus (*r* = −0.442 and *r* = −0.389, resp.). Correlations between mononuclear cell infiltration and PG levels have already been reported [8, 10]. We decided that scrutinizing mononuclear cell infiltration as a parameter in assessing chronic histological changes would be worthwhile, classified histological gastritis based on the distribution pattern of mononuclear cell infiltration in the gastric mucosa, and analyzed the correlation to serum PG levels. Results of ROC analysis revealed that progression of histological gastritis as defined by the distribution of mononuclear cell infiltration can be predicted relatively well using serum PG I/II ratio. The optimal PG I/II ratio cutoff for distinguishing between absence of histological gastritis (N group) and presence of histological gastritis (A, C, and P groups) was 4.0, and the optimal cutoff for distinguishing between normal or histological gastritis limited to the antrum (N and A groups) and histological gastritis that had progressed to the corpus (C and P groups) was 3.2. 

**Figure 6:**
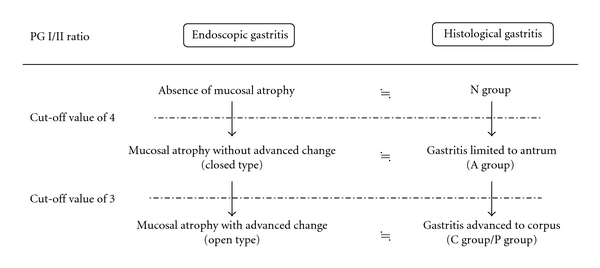
Correlation between endoscopic gastric mucosal atrophy and histological gastritis with PG I/II ratio. Setting the respective cut-off values to about 4 and 3 appeared to have enabled estimation of the presence or absence of endoscopic atrophy and histological gastritis, or the extent of their development.

Examination of the correlation between PG levels and endoscopic gastric mucosal atrophy based on the Kimura-Takemoto classification revealed an optimal PG I/II ratio cutoff of 3.9 for distinguishing between absence of endoscopic gastric mucosal atrophy and presence of gastric mucosal atrophy (closed and open types), and an optimal cutoff of 2.9 for distinguishing between cases without advanced atrophy (no gastric mucosal atrophy, closed type) and cases with advanced atrophy (open type). These values are generally consistent with the optimal cut-off values for distinguishing between absence of histological gastritis and presence of histological gastritis, and between normal or histological gastritis limited to the antrum and histological gastritis that has advanced to the corpus. Setting the respective cut-off values to about 4 and 3 appeared to have enabled estimation of the presence or absence of endoscopic atrophy and histological gastritis or the extent of their development. Based on the present analysis of the same population, PG I/II ratio appears useful for predicting the extent to which endoscopic gastritis and histological gastritis have advanced, and, from the perspective of PG I/II ratio, histological gastritis limited to the antrum (A group) and endoscopic gastritis without advanced atrophic changes (closed type) correspond to each other, as do histological gastritis that has advanced to the gastric corpus (C group/P group) and endoscopic gastritis with advanced atrophic changes (open type) (Figure 6). These findings from this study could contribute to shared understanding of chronic gastritis that various doctors such as gastrointestinal endoscopist and pathologist evaluated by three different methods, that is, endoscopy, microscopy, or PG test.

The Sydney system was proposed, and the description and classification of histological gastritis have become fairly well established, whereas no international consensus has yet been reached on the description and classification of endoscopic gastritis. Points of correspondence between histological gastritis based on the updated Sydney classification and endoscopic gastritis based on the Kimura-Takemoto classification have also not been studied in great deal. The present study of correlations between endoscopic gastritis (Kimura-Takemoto classification) and histological gastritis (updated Sydney classification), including serum PG levels, in the same population showed strong correlations and points of correspondence between the three systems. This represents the first attempt at a multifaceted look at chronic gastritis from serological, endoscopic, and histological perspectives to ascertain correlations and points of correspondence, and the results here may prove clinically useful in contemplating chronic gastritis.

## Figures and Tables

**Table 1:** Clinical characteristics of patients.

Age (years)	56.5 (12–94)
Sex: male/female	200/119
Hp seropositive (%)	69.0 (220/319)
Endoscopic findings	
Normal	11
Atrophic gastritis	258
Gastric ulcer	53
Duodenal ulcer	39
Erosive gastritis	17
Gastric cancer	39
Serum pepsinogens	
Pepsinogen I	71.99 (38.65–80.90)*
Pepsinogen II	22.37 (11.48–26.63)*
Pepsinogen I/II ratio	3.55 (2.20–4.70)*
Kimura-Takemoto classification	
Absence of any atrophy	60
Closed-type gastritis	146
Open-type gastritis	113

*Variables are presented as median (interquartile range) for skewes variables.

**Table 2:** Pepsinogen I/II ratio in relation to histopathology scores.

	*r*	*P*
Mono		
Antrum	−0.309	< 0.0001
Corpus	−0.389	< 0.0001
Neutro		
Antrum	−0.256	0.0007
Corpus	−0.442	<0.0001
Atrophy		
Antrum	−0.222	0.0044
Corpus	−0.293	0.0001
Hp		
Antrum	−0.174	0.022
Corpus	−0.291	<0.0001
IM		
Antrum	−0.218	0.004
Corpus	−0.211	0.0054

Mono = mononuclear cells.

Neutro: neutrophils.

Hp: *Helicobacter pylori.*

IM: intestinal metaplasia.

**Table 3:** Classification of histological gastritis based on mononuclear cell infiltration.

Corpus Degree of mononuclear cells	Antrum degree of mononuclear cells	
	0 or 1 (absent or mild)	2 or 3 (moderate or severe)

0 or 1	N	A
(absent or mild)	(37.1%)	(84.4%)

2 or 3	C	P
(moderate or severe)	(78.3%)	(86.7%)

N: normal or mild gastritis (serum Hp antibody-positive rate).

A: antrum predominant gastritis.

C: corpus predominant gastritis.

P: pangastritis.

**Table 4:** Endoscopic gastric mucosal atrophy scores in relation to histopathology scores.

	*r*	*P *
Mono		
Antrum	0.184	0.0169
Corpus	0.459	<0.0001
Neutro		
Antrum	0.036	0.6422
Corpus	0.382	<0.0001
Atrophy		
Antrum	0.185	0.0195
Corpus	0.390	<0.0001
Hp		
Antrum	−0.06	0.4437
Corpus	0.156	0.0427
IM		
Antrum	0.348	<0.0001
Corpus	0.196	0.0108

Mono = mononuclear cells.

Neutro = Neutrophils.

Hp = *Helicobacter pylori*.

IM = intestinal metaplasia.
